# Prevalence and Genetic Diversity of *Echinococcus granulosus* Sensu Stricto in Sheep from Kazakhstan

**DOI:** 10.3390/biology15100779

**Published:** 2026-05-14

**Authors:** Rabiga Uakhit, Aidana Tautanova, Ainura Smagulova, Carlos Hermosilla, Aida Abdybekova, Lyudmila Lider, Karina Jazina, Marat Dusmagambetov, Vladimir Kiyan

**Affiliations:** 1 Laboratory of Biodiversity and Genetic Resources, National Center for Biotechnology, 010000 Astana, Kazakhstan; erken.uakhitrabiga@gmail.com (R.U.); smagulova0114@gmail.com (A.S.); aida_abdybekova@mail.ru (A.A.); l.lider@kazatu.kz (L.L.); dzhazinak01@mail.ru (K.J.); 2 Department of Microbiology and Virology, Astana Medical University, 010000 Astana, Kazakhstan; tautanovaa@mail.ru (A.T.); dusmagambetov.m@amu.kz (M.D.); 3 Institute of Parasitology, Justus Liebig University Giessen, 35392 Giessen, Germany; carlos.r.hermosilla@vetmed.uni-giessen.de; 4 Laboratory of Parasitology and Mycology, Kazakh Scientific Research Veterinary Institute, 050016 Almaty, Kazakhstan; 5 Department of Veterinary Medicine, S. Seifullin Kazakh Agrotechnical Research University, 010000 Astana, Kazakhstan; 6 Scientific Center for Biological Research, 010000 Astana, Kazakhstan

**Keywords:** cystic echinococcosis, genetic diversity, Kazakhstan, zoonotic disease, genotype G1, haplotype analysis, livestock management

## Abstract

Cystic echinococcosis (CE) is a zoonotic disease caused by the larval stage of the *Echinococcus granulosus* sensu lato (s.l.) complex. The disease is globally distributed, with particularly high prevalence in Central Asian countries, including Kazakhstan. Despite its significant impact on public health and livestock production, data on CE in sheep in Kazakhstan remain limited. This study investigated the prevalence and genetic diversity of *Echinococcus granulosus* sensu stricto (s.s.) in sheep across Kazakhstan, addressing an important zoonotic disease affecting both livestock and human health. Over the course of one year, a total of 31,389 sheep were examined, and cystic echinococcosis cysts were collected from the livers and lungs of 550 infected sheep across 14 regions of Kazakhstan. Molecular analyses targeting mitochondrial genes (*nad1*, *cox1*) were performed to determine genetic diversity. The results revealed a higher occurrence of CE in the southern regions of the country. Among the genotyped isolates (57), genotype G1 was dominant, accounting for 84.2% (48) of the samples, whereas genotype G3 (9) was detected at a lower frequency in three regions. A total of 11 distinct haplotypes were identified, indicating considerable genetic diversity among the isolates. Haplotype network analysis suggested gene flow among populations and revealed the widespread presence of the most common haplotype (EgKZ-2) across multiple regions. These findings highlight the need for continuous monitoring and targeted control strategies for cystic echinococcosis, emphasizing the importance of understanding parasite genetic diversity for public health interventions and livestock management in endemic areas. Overall, this study contributes to the understanding of the genetic diversity and transmission dynamics of *E. granulosus* s.s. in Central Asia.

## Simple Summary

Cystic echinococcosis is a parasitic disease that affects both animals and humans and remains a major health and economic problem in many parts of the world, including Kazakhstan. Sheep are one of the main intermediate hosts of the parasite and play an important role in its transmission. However, information on the occurrence and genetic diversity of the parasite in sheep in Kazakhstan is limited. In this study, we investigated the presence of the parasite in sheep from different regions of the country and analyzed its genetic characteristics. The results showed that the disease is more common in southern regions and that the G1 genotype is the dominant strain circulating in sheep populations. Understanding the genetic diversity and distribution of this parasite can help improve monitoring and develop more effective control strategies to reduce the spread of cystic echinococcosis.

## 1. Introduction

*Echinococcus* is a genus of parasitic tapeworms, with several species responsible for cystic echinococcosis, a zoonotic disease that poses significant public health and veterinary concerns globally. Among them, the *Echinococcus granulosus* sensu lato (s.l.) complex is particularly important because of its global distribution and its role as the causative agent of echinococcosis [1,2]. Cystic echinococcosis (CE) affects a wide range of intermediate hosts, including livestock and humans, and leads to substantial economic losses due to decreased livestock productivity and costs associated with medical treatment and disease control [1,3,4]. The disease CE is considered one of the most important neglected zoonotic infections in many pastoral and rural regions of the world [3,5].

The *E. granulosus* complex comprises several species and genotypes with different host preferences and epidemiological characteristics. Within this complex, *E. granulosus* sensu stricto (s.s.) is the most widely distributed and epidemiologically important species and corresponds to isolates historically classified as genotypes G1–G3 [2,6]. Other members of the complex include *Echinococcus canadensis* (G6–G7 genotypes) and *Echinococcus ortleppi* (G5 genotype), which contribute to the genetic and epidemiological diversity of the parasite [6,7]. The G1 genotype, historically referred to as the “sheep strain,” predominantly circulates in sheep and goats as intermediate hosts and is the principal cause of human CE worldwide. This strain is also recognized as the most frequent cause of human CE worldwide [8,9,10].

The life cycle of *Echinococcus* species typically involves canids, mainly domestic dogs, as definitive hosts, while livestock such as sheep, goats, and cattle serve as intermediate hosts [11]. Infected definitive hosts shed parasite eggs in their feces, contaminating pastures, water sources, and the environment. Intermediate hosts become infected through ingestion of these eggs, which then develop into cysticechinococcosis cysts in internal organs [12,13]. Humans can accidentally become intermediate hosts after ingesting parasite eggs through contaminated food, water, or direct contact with infected dogs. The close interaction between domestic animals, dogs, and humans in pastoral and rural communities facilitates the maintenance of the parasite life cycle and increases the risk of zoonotic transmission [14].

Over the past decades, molecular techniques have greatly improved the understanding of the taxonomy, genetic diversity, and epidemiology of *Echinococcus* species. Polymerase chain reaction (PCR)-based methods combined with DNA sequencing allow precise identification of parasite genotypes and help clarify transmission patterns in different geographical regions [15]. Mitochondrial markers are particularly useful for studying the population genetics of *Echinococcus*. Among them, the cytochrome c oxidase subunit 1 (*cox1*) and NADH dehydrogenase subunit 1 (*nad1*) genes are widely used due to their high variability and ability to distinguish closely related genotypes [16,17]. Analysis of these genes provides valuable information about parasite genetic diversity, evolutionary relationships, and patterns of parasite spread [18].

Cystic echinococcosis remains a significant public health and veterinary problem in many regions of Central Asia, including Kazakhstan, Kyrgyzstan, Uzbekistan, and Turkmenistan, as well as in parts of Russia and other Asian countries [19,20,21,22,23]. Livestock farming plays an important role in the economies of these regions, and traditional animal husbandry practices often involve close contact between livestock, dogs, and humans. Under these conditions, the transmission cycle of *Echinococcus* parasites can be easily maintained, contributing to the persistence of the disease in both animal and human populations [24,25]. Kazakhstan, with its extensive sheep farming (approximately 19.1 million head) and large rural areas [26,27], represents an important region for studying the epidemiology of cystic echinococcosis.

Although cystic echinococcosis is widespread in Kazakhstan [28], information on the prevalence and genetic diversity of *E. granulosus* sensu stricto circulating in livestock remains limited. Most available studies have focused on clinical cases in humans or general epidemiological surveys, while molecular data on parasite genotypes in sheep are still scarce [20,29]. Sheep were selected for the present study because they represent the principal intermediate host for *E. granulosus* sensu stricto (particularly the G1 genotype) and play a central role in maintaining transmission within the dog–livestock cycle. Focusing on this host species therefore provides epidemiologically relevant insights into the dominant transmission pathway and the genetic structure of parasite populations in the region. Understanding the genetic structure and geographical distribution of parasite populations is essential for assessing zoonotic risk and developing effective disease control strategies.

Therefore, this study aimed to investigate the prevalence and genetic diversity of *E. granulosus* sensu stricto in sheep from different regions of Kazakhstan. Specifically, mitochondrial *nad1* and *cox1* gene sequences were analyzed using PCR amplification and sequencing to identify circulating genotypes and evaluate the genetic diversity of parasite populations.

## 2. Materials and Methods

### 2.1. Sample Collection

**Figure 1:**
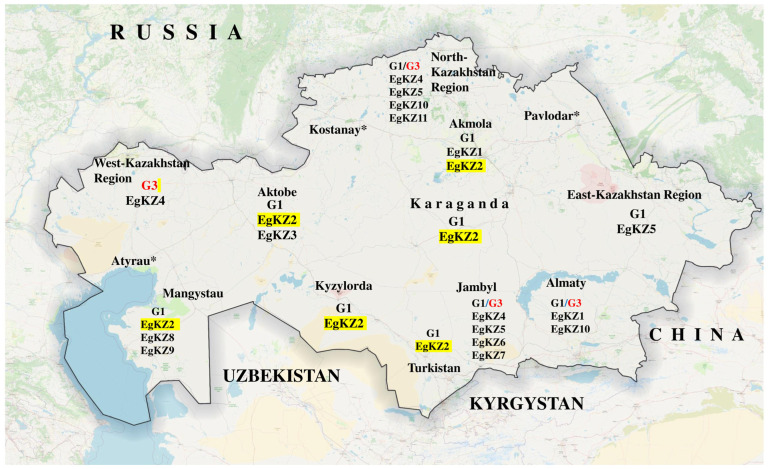
Locations where the sheep isolates originated in the study, along with their assigned genotypes and *cox1* gene haplotypes detected. * Region in which there were no confirmed cases of ovine cystic echinococcosis (CE) during the sample collection period.

This cross-sectional study was conducted from January to December 2022 and included 31,389 sheep examined at officially registered slaughterhouses and veterinary meat inspection facilities across 14 administrative regions of Kazakhstan, as presented in Figure 1 (Kostanay, Akmola, Turkistan, Pavlodar, Karaganda, East Kazakhstan, West Kazakhstan, Aktobe, Atyrau, North Kazakhstan, Almaty, Kyzylorda, Jambyl, and Mangystau). During routine post-mortem inspection, internal organs, primarily the liver and lungs, were subjected to systematic visual examination, palpation, and deep longitudinal incision by certified veterinary inspectors and parasitology specialists to detect cystic echinococcosis cysts. Infected organs (lungs and liver) containing cystic echinococcosis cysts were identified in 550 sheep, from each of which one cyst was collected for further genetic analysis.

The cyst samples originated from sheep examined in different regions of Kazakhstan during routine slaughterhouse surveillance. However, because organ-specific metadata were not consistently documented at all collection sites, retrospective assignment of the exact organ source was not possible for all samples. This limitation did not affect the molecular genotyping objectives of the study, as all selected samples consisted of intact cystic echinococcosis cyst material suitable for DNA-based species identification.

**Figure 2:**
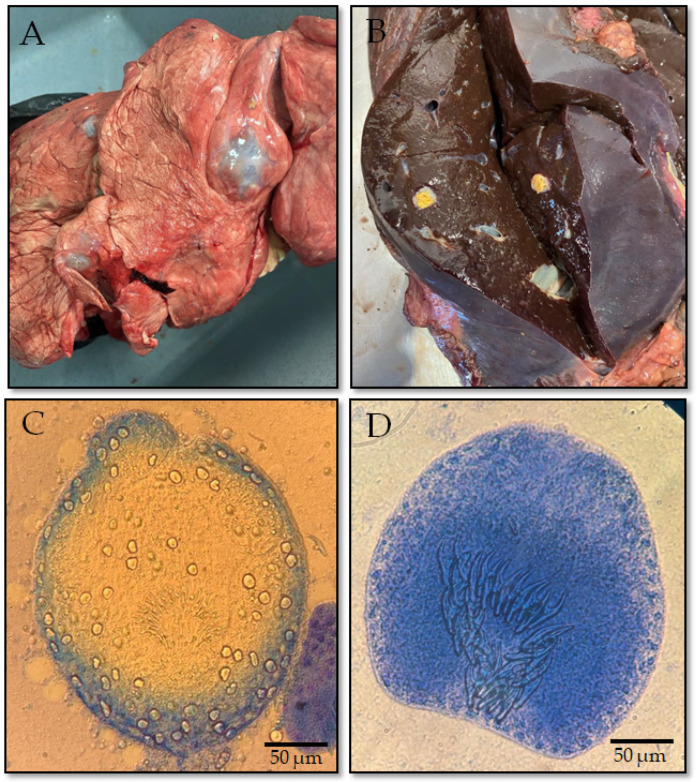
Multiple cystic echinococcosis (CE) cysts in the lungs (**A**) and liver (**B**) of a sheep, and viable (**C**) and non-viable (**D**) protoscolex of *Echinococcus granulosus*.

Macroscopic examination was carried out by certified veterinary inspectors and parasitology specialists as part of standard postmortem meat inspection procedures. Internal organs, primarily liver and lungs, were visually inspected, palpated, and systematically incised to detect CE cysts (Figure 2A,B). Cysts were classified according to organ localization and morphological characteristics [2]. A sample was considered positive for CE when at least one CE cyst morphologically consistent with infection by *E. granulosus* sensu lato was detected during post-mortem examination. Fertility status was assessed by microscopic examination of cyst fluid for the presence of protoscoleces (Figure 2). Protoscolex viability was assessed using 0.4% trypan blue exclusion staining under light microscopy. Viable protoscoleces excluded the dye, while non-viable ones were stained blue. Only intact cyst material suitable for molecular analysis was included in the genetic study. For molecular confirmation and genotyping, protoscoleces or germinal layers were aseptically collected, preserved in 70–96% ethanol, and stored at −20 °C until DNA extraction.

The sample flow from selected CE cysts is as follows: infected organ–cyst–germinal layer collection–DNA extraction–molecular-genetic analysis–bioinformatic analysis–statistical analysis.

### 2.2. DNA Extraction Method

The cysts exhibited variable pathological conditions, including calcified and purulent forms. Therefore, 68 intact cysts with preserved germinal layer material and adequate tissue quality were selected for subsequent molecular characterization. DNA was effectively extracted from 57 cysts using a Genomic DNA Purification kit (Monarch^®^, Cat.: T3010L, Ipswich, MA, USA) following the manufacturer’s recommendations with some minor adjustments. Briefly, cyst material obtained from infected organs (lungs and livers) was placed into Eppendorf tubes and homogenized using a sterile pestle in the presence of tissue lysis buffer and Proteinase K. The homogenized samples were incubated to ensure complete tissue digestion. Subsequent DNA extraction steps were performed according to the manufacturer’s protocol for the kit. DNA was finally eluted in 1×TE buffer and stored at −20 °C until further analysis. DNA concentration and purity were measured using a NanoDrop™ (Thermo Fisher Scientific, Waltham, MA, USA).

### 2.3. PCR Analysis and Sequencing

Polymerase chain reaction (PCR) was used to assess the genetic diversity of *Echinococcus* spp. with two primer pairs targeting cytochrome c oxidase subunit 1 (*cox1*: forward 5′-TTTTTTGGGCATCCTGAGGTTTAT-3′ and reverse 5′-TAAAGAAAGAACATAATGAAAATG-3′) and dehydrogenase subunit 1 (*nad1*: forward 5′-TGGAACTCAGTTTGAGCTTTACTA-3′ and reverse 5′-ATATCAAAGTAACCTGCTATGCAG-3′) [30,31]. PCR was carried out in a 25 μL reaction mixture containing 2× DreamTaq PCR Master Mix (Thermo Fisher Scientific), nuclease-free water, 10 pmol of each primer, and 20 ng of genomic DNA. The cycling conditions included an initial denaturation at 94 °C for 10 min, followed by 35 cycles of denaturation at 94 °C (30 s), annealing at 45 °C (45 s), and extension at 72 °C (1 min), with a final extension at 72 °C for 7 min. The PCR products were separated on a 1.0% agarose gel with 1× TBE buffer, stained with ethidium bromide (8 ng/μL), and visualized under UV light.

All PCR-positive products were purified with the Quick PCR Purification Kit (QIAGEN, Germantown, MD, USA) following the manufacturer’s instructions. They were then processed for sequencing and genotyping. Sequencing was conducted using a 3730xl DNA Analyzer 96-Capillary Array (Thermo Fisher Scientific, Applied Biosystems, Foster City, CA, USA). The genetic sequences generated in this study are deposited in GenBank under BioProject PRJNA1131194. These nucleotide sequences were manually refined and compared to reference sequences from GenBank using the BLAST algorithm (https://www.ncbi.nlm.nih.gov/, accessed on 13 March 2026).

### 2.4. Alignment and Phylogenetic, Haplotype Analysis of Mitochondrial Genes cox1 and nad1

The sequences obtained were manually edited. Sequence similarity searches were performed against the GenBank reference sequences using the BLAST algorithm (https://blast.ncbi.nlm.nih.gov, accessed on 13 March 2026). Nucleotide sequences for *cox1* and *nad1* partial genes were aligned with the MUSCLE multiple sequence alignment program. All obtained sequences were deposited in the GenBank database and assigned the following accession numbers: PQ177868–PQ177870, PQ045256–PQ045261, PQ002233–PQ002236, and PQ002207–PQ002210. Phylogenetic trees were built using a concatenated dataset in MEGA11 software, employing the Maximum Likelihood (ML) method [32]. For phylogenetic comparison, representative external *cox1* reference sequences were retrieved from GenBank to cover the principal genotypes within the *E. granulosus* sensu lato complex. Reference sequences of *E. granulosus* sensu stricto genotype G1 included PV584259, PV584258, and PV584261, whereas genotype G3 was represented by HM563022, PV584256, and PV584257. Additional comparison genotypes outside the *E. granulosus* s.s. cluster included *E. equinus* genotype G4 (KM014645, JX068641, KM525658), *E. intermedius* genotype G6 (MT227304, OQ255722, OQ255723), *E. canadensis* genotype G8 (AB777910, MH828449), and *E. canadensis* genotype G10 (OR420701, OR420702, OR420703). The cestode *Taenia hydatigena* (MZ345598) was used as an outgroup to root the tree. Population diversity indices calculated included nucleotide diversity (πd), haplotype diversity (hd), and haplotype number (hn). Neutrality tests included Tajima’s D [33], Fu’s statistics and Fu’s Fs [34], Fu and Li’s D test (FLD), and Fu and Li’s F (FLF) statistics. Parsimony-informative analysis was conducted using DnaSP6, and a haplotype network was constructed with Popart v1.7 [35].

### 2.5. Parasitological Statistical Analysis

Prevalence was calculated as the proportion of infected animals among the examined population with 95% confidence intervals. Differences between regions were assessed using the Pearson chi-square (χ^2^) test. The distribution of cysticechinococcosis cyst counts among regions was also evaluated using a chi-square goodness-of-fit test. A *p*-value < 0.05 was considered statistically significant [36,37].

## 3. Results

### 3.1. Parasitological Research

During postmortem examination, 31,389 sheep were inspected for ovine CE. A total of 550 infected sheep were identified, and one CE cyst was collected from each animal: 65 from the South Kazakhstan (Turkistan) Region, 50 from Kyzylorda, 51 from Jambyl, 54 from Almaty, 55 from Akmola, 46 from North Kazakhstan, 50 from East Kazakhstan, 41 from Mangystau, 34 from Aktobe, 49 from West Kazakhstan, and 55 from Karagandy.

Among the 550 collected CE cyst samples, cysts were predominantly localized in the liver and lungs. Microscopic examination demonstrated that 64.8% of the examined cysts were fertile and contained protoscoleces. Viability assessment using trypan blue exclusion staining showed a mean protoscolex viability of 74.6%, confirming the suitability of the collected material for subsequent molecular analysis.

The gross post-mortem animal-level prevalence of cystic echinococcosis was 1.7% (95% CI: 1.6–1.9; 550/31,389 sheep), based on the detection of at least one morphologically consistent hydatid cyst during routine slaughter inspection. Significant difference in prevalence among regions was observed (χ^2^ = 31.62, df = 10, *p* < 0.001). The regional distribution of cystic echinococcosis cysts also differed significantly across the studied regions (χ^2^ = 103.27, df = 10, *p* < 0.001).

Over the one-year study period, the highest number of CE cysts was detected in the South Kazakhstan region, whereas no cases of echinococcosis in sheep were reported in Atyrau, Kostanay, or Pavlodar regions during the study period (Table 1).

### 3.2. Molecular Genetics Identification

**Figure 3:**
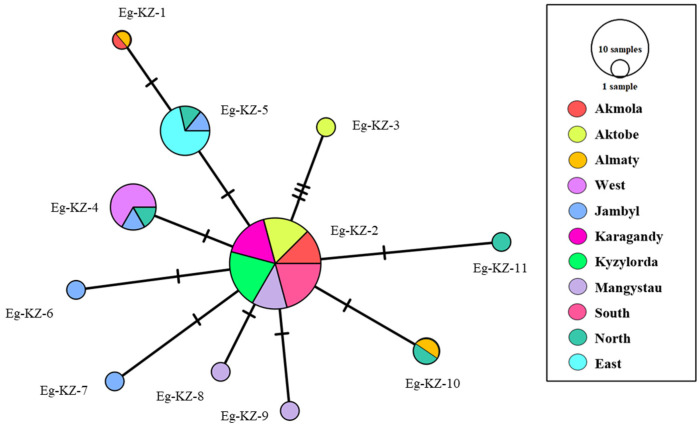
The haplotype network for the concatenated mitochondrial genes *cox1* and *nad1* of *Echinococcus granulosus* s.s. Circles depict various haplotypes, with their sizes proportional to haplotype frequency. Colors denote geographic origin. Haplotypes identified in this study are labeled as Eg-Kz-n, where Eg indicates *Echinococcus granulosus*, KZ refers to Kazakhstan, and n represents the haplotype number.

Based on the obtained data, haplotype and genotype analyses were carried out on the isolated DNA using the sequencing method for mitochondrial regions of the genome. The *cox1* primers produce a ~440 bp fragment with a readable length of approximately ~300 bp. The *nad1* gene amplifies a ~530 bp fragment, successfully yielding a readable sequence of approximately ~450 bp after primer removal. In the final dataset, the haplotype network was retrieved based on concatenated *cox1* and *nad1* sequences that included 750 bp. Haplotype diversity analysis revealed 11 distinct haplotypes among 47 isolates from 11 regions (Figure 3).

Thus, 11 haplotypes were identified among the studied isolates from 11 regions. The analysis does not include the three regions mentioned above in which no cases of ovine CE were detected during the sample collection period. The analysis shows that EgKZ-2 is the most common in Kazakhstan among the studied sheep samples. It should also be noted that there are haplotypes consisting of single isolates. A haplotype network based on TCS analysis is shown in Figure 3. In the network, EgKZ-1 and EgKZ-3 appear relatively isolated and are separated from other haplotypes by several mutational steps, which may suggest some genetic differentiation. In Table 2, data on diversity and neutrality indices.

Tajima’s D value indicated a population buildup and/or refinement of selection, while the negative Fu’s Fs value suggested the presence of uncommon haplotypes resulting from recent population expansion or hitchhiking. These findings are further supported by the fact that 63.6% (6 out of 11) of the haplotype groups consisted of single haplotypes. A total of 11 haplotypes were identified. The most common haplotype is EgKZ-2, which was found in 25 isolates from 10 regions. This indicates a widespread presence of this haplotype; EgKZ-5 and EgKZ-7 were the next most common.

**Figure 4:**
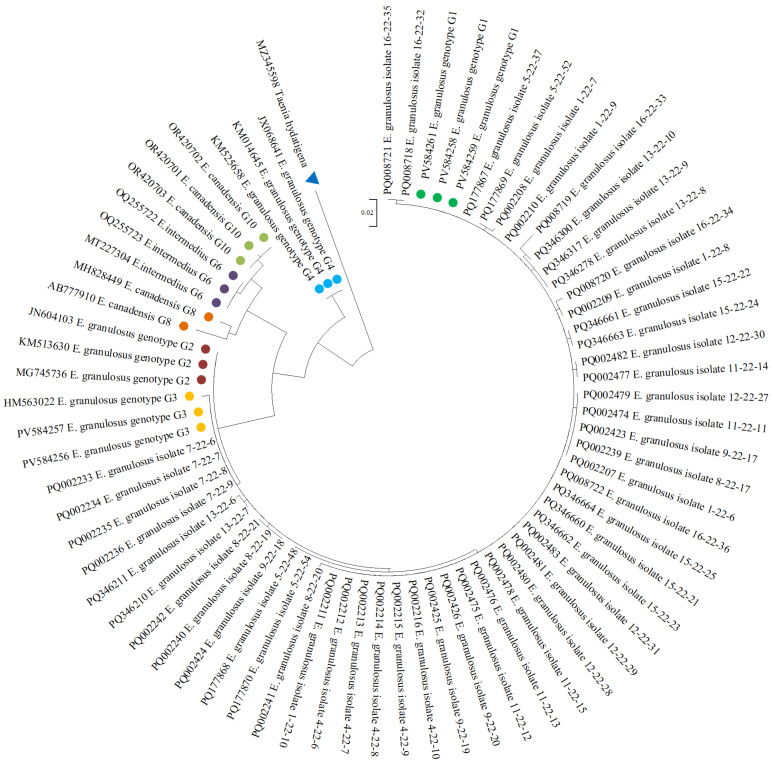
Maximum-likelihood phylogenetic tree built from *cox1* gene sequences of *Echinococcus granulosus* sensu stricto isolates. The blue triangle indicates the outgroup (*Taenia hydatigena*, MZ345598).

Based on *cox1* sequences, a maximum likelihood phylogenetic tree was constructed (Figure 4). The tree was constructed from GenBank sequences of the studied isolates. MZ345598 *Taenia hydatigena* isolate was used as outgroup to root the tree.

Phylogenetic relationships among isolates of *E*. *granulosus* were inferred based on the mitochondrial *cox1* gene. Sequence alignment was performed using the MUSCLE algorithm, and the phylogenetic tree was reconstructed using the Maximum Likelihood (ML) method. The cestode *T*. *hydatigena* was used as an outgroup to root the tree.

The ML phylogenetic analysis showed that the majority of isolates from the present study clustered within the G1 genotype clade, forming a well-supported monophyletic group together with reference sequences of the sheep strain. These isolates grouped tightly with previously published G1 sequences (PV584259, PV584258, PV584261), indicating high genetic similarity among them. Also, isolates were positioned within the G3 (HM563022, PV584256, PV584257) genotype clade, clustering with reference sequences representing the buffalo strain. The G1 and G3 genotypes formed a larger monophyletic cluster corresponding to *E. granulosus* s.s. Bootstrap support values for the major nodes ranged from 85% to 90%, indicating strong phylogenetic support.

Other genotypes included for comparison formed distinct clades outside the *E. granulosus* s.s. group. These included *E. canadensis* genotype G8 (AB777910, MH828449), *E. intermedius* genotype G6 (MT227304, OQ255722, OQ255723), *E. canadensis* genotype G10 (OR420701, OR420702, OR420703), and *E. equinus* genotype G4 (KM014645, JX068641, KM525658), each clearly separated from the G1–G3 cluster.

## 4. Discussion

The study provides insights into the genotypes, haplotypes, and distribution of *E. granulosus* s.s. in sheep in Kazakhstan. Understanding the genetic diversity of this parasite is crucial for assessing zoonotic risk and informing targeted control programs.Identification of different genotypes and haplotypes contributes to a better understanding of the epidemiology and geographic distribution of *E. granulosus* s.s. in sheep and other intermediate hosts, which is important for designing effective control strategies [7,38,39]. Our findings represent one of the first large-scale assessments of molecular diversity of *E. granulosus* s.s. across multiple regions of Kazakhstan, highlighting its relevance for both livestock management and public health. This study also provides a baseline for future monitoring of parasite genetic diversity in Central Asia, which is essential for predicting changes in transmission dynamics and potential emergence of new genotypes [6,11,17].

The results demonstrated that CE in sheep was detected in several regions, with the highest prevalence observed in South Kazakhstan (2.7%), followed by Akmola (2.4%) and Almaty regions (2.3%). Lower infection rates were recorded in Mangystau (1.8%) and Aktobe (1.5%), while no cases were detected in Atyrau, Kostanay, and Pavlodar regions. Statistical analysis revealed significant differences in infection prevalence among the studied regions (χ^2^ = 31.62, df = 10, *p* < 0.001). Similarly, the distribution of CE cyst numbers differed significantly between regions (χ^2^ = 103.27, df = 10, *p* < 0.001), indicating heterogeneous spatial distribution of the parasite.

The haplotype network analysis revealed the genetic structure and diversity among the studied CE isolates. The most frequent haplotype is EgKZ-2, which includes samples from multiple regions (Figure 3). This suggests that EgKZ-2 is a common genetic variant shared across different geographical areas in Kazakhstan, possibly due to historical gene flow or shared ancestry. The presence of multiple regions within this haplotype indicates a widespread distribution of this genetic type, making it a central and possibly ancestral haplotype in the network. The high prevalence of EgKZ-2 across geographically distinct regions may reflect livestock movements, trade practices, and shared pastures between neighboring regions, contributing to parasite dispersal [17,18].

The observed values of Tajima’s D (−1.9089) and Fu’s Fs (−7.133) support the idea of population expansion and selection. Tajima’s D being negative suggests an excess of low-frequency polymorphisms, which is a pattern often associated with population growth or selective sweeps. Similarly, a negative Fu’s Fs indicates an excess of rare haplotypes, reinforcing the idea of recent population expansion and the emergence of unique haplotypes. In our analysis, nine previously undescribed *cox1* haplotypes were identified. The discovery of novel haplotypes highlights ongoing genetic evolution in the parasite population and suggests the presence of region-specific variants that may affect transmission dynamics [9,40].

The most common *cox1* haplotype infecting sheep in Kazakhstan is EgKZ-2. The haplotype network indicates a genetic landscape shaped by population expansion and the emergence of unique haplotypes in Kazakhstan. This diversity underscores the dynamic nature of the genetic evolution within the population studied [38].These patterns may also reflect historical livestock movement across Central Asia and the ongoing role of transhumance in shaping parasite gene flow, consistent with findings from neighboring regions [19,22].

With the highest detection rate, genotype G1 is dominant and responsible for most infections in Kazakhstan. The dominance of G1 is consistent with its widespread role in human cystic echinococcosis globally [6,7,8] and highlights sheep as the primary intermediate host in Kazakhstan. The restricted distribution of G3 suggests either recent introduction or localized transmission, which may be influenced by regional husbandry practices and movement of livestock between specific regions [12,13]. This genotype-specific prevalence data supports targeted interventions, as control measures might focus first on G1-dominated populations to maximize public health impact.

In Central Asia, the prevention and control of human CE remain significant public health challenges [31,41]. Our findings are in line with reports from neighboring regions of Central Asia, particularly in China where genotype G1 accounts for the vast majority of *E. granulosus* s.s. infections in livestock and humans [42,43,44,45,46], and in Uzbekistan, where G1 is also frequent among human and sheep isolates [47]. Additionally, the regional distribution patterns of haplotypes in Kazakhstan suggest that livestock movement, shared pastures, and local husbandry practices contribute substantially to parasite dispersal and the maintenance of transmission cycles [20,21,29].

The widespread presence of dominant haplotypes and genotypes indicates active parasite transmission across Kazakhstan, facilitated by traditional livestock management and dog–sheep interactions. Control strategies should focus on integrated approaches: regular deworming of dogs, improved slaughterhouse hygiene, restriction of home slaughter, and public health education, following the “One Health” framework [5,25,29,48].

Although 31,389 sheep from 14 regions of Kazakhstan were examined, the sampling may not fully represent the entire sheep population of the country. Only 550 CE cysts were collected, of which 57 were successfully analyzed molecularly, potentially limiting the detection of rare haplotypes. Some cysts were calcified or purulent, which could affect DNA extraction efficiency and bias the identification of unique haplotypes. Limited information on age, breed, and management practices of sampled sheep constrains the assessment of epidemiological risk factors for infection. Furthermore, some remote or border regions with low livestock density were not included, which may affect the generalizability of genotype and haplotype distribution across Kazakhstan.

## 5. Conclusions

The data on the prevalence of sheep echinococcosis in Kazakhstan across different regions are crucial for understanding the distribution of this parasitic infection. The study’s findings highlight the genotypic diversity and distribution of *E. granulosus* s.s. in sheep in Kazakhstan and contribute to a better understanding of the epidemiology of ovine CE in the region. Variation in genotypes and haplotypes may reflect differences in transmission patterns and may be relevant for epidemiological studies, diagnostic development, and future vaccine research. Overall, these insights can inform public health interventions for the prevention and management of CE in Kazakhstan and other regions facing similar challenges.

## Figures and Tables

**Table 1:** Prevalence of cystic echinococcosis (CE) in slaughtered sheep in different regions of Kazakhstan.

Region	Number of Sheep Examined	Count of Infected Sheep (95% Confidence Interval)	Chi-Square, *p*-Value	Count of Observed CE Cysts	Chi- Square, *p*-Value
Turkistan	2384	65 (2.7)	χ^2^ = 31.62, df = 10, *p* < 0.001	325	χ^2^ = 103.27, df = 10, *p* < 0.001
Kyzylorda	2329	50 (2.1)		252	
Jambyl	2327	51 (2.2)		249	
Almaty	2338	54 (2.3)		274	
Akmola	2246	55 (2.4)		273	
North Kazakhstan	2233	46 (2.1)		231	
East Kazakhstan	2240	50 (2.2)		247	
Karagandy	2342	55 (2.3)		264	
Mangystau	2202	41 (1.9)		204	
Aktobe	2187	34 (1.5)		172	
West Kazakhstan	2054	49 (2.4)		145	
Atyrau	2182	-		-	
Kostanay	2167	-		-	
Pavlodar	2158	-		-	
Total	31,389	550 (1.7)		2636	

df—degrees of freedom; χ^2^—Chi-square.

**Table 2:** Diversity and neutrality indices were obtained using concatenated nucleotide data of the *Echinococcus granulosus* s.s.

n	hn	hd ± SD	Tajima’s D	*p* Value	Fu’s Fs	*p* Value	FLD	*p* Value	FLF	*p* Value
47	11	0.688 ± 0.066	−1.9089	*p* < 0.05	−7.133	0.001	−3.3113	*p* < 0.05	−3.1624	*p* < 0.02

n: number of isolates; hn: number of haplotypes; hd: haplotype diversity; SD: standard deviation; FLD: Fu and Li’s D test statistic; FLF: Fu and Li’s F statistics test.

## Data Availability

The original contributions presented in this study are included in the article. Further inquiries can be directed to the corresponding author.

## Abbreviations

The following abbreviations are used in this manuscript:

bp	base pairs
CE	cystic echinococcosis
*cox1*	cytochrome c oxidase subunit 1
DNA	deoxyribonucleic acid
*nad1*	NADH dehydrogenase subunit 1 ()
s.l.	sensu lato
s.s.	sensu stricto
TBE	Tris-borate-EDTA buffer
UV	ultraviolet
